# Automatic diagnosis of Parkinson’s disease using artificial intelligence base on routine T1-weighted MRI

**DOI:** 10.3389/fmed.2023.1303501

**Published:** 2024-01-05

**Authors:** Chang Li, Dongming Hui, Faqi Wu, Yuwei Xia, Feng Shi, Mingguang Yang, Jinrui Zhang, Chao Peng, Junbang Feng, Chuanming Li

**Affiliations:** ^1^Bioengineering College of Chongqing University, Chongqing University Central Hospital (Chongqing Emergency Medical Center), Chongqing, China; ^2^Department of Radiology, Chongqing Western Hospital, Chongqing, China; ^3^Department of Medical Service, Yanzhuang Central Hospital of Gangcheng District, Chongqing, China; ^4^Department of Research and Development, Shanghai United Imaging Intelligence, Co., Ltd. Shanghai, China

**Keywords:** PD, radiomics, artificial intelligence, T1-weighted, MRI

## Abstract

**Background:**

Parkinson’s disease (PD) is the second most common neurodegenerative disease. An objective diagnosis method is urgently needed in clinical practice. In this study, deep learning and radiomics techniques were studied to automatically diagnose PD from healthy controls (HCs).

**Methods:**

155 PD patients and 154 HCs were randomly divided into a training set (246 patients) and a testing set (63 patients). The brain subregions identification and segmentation were automatically performed with a VB-net, and radiomics features of billateral thalamus, caudatum, putamen and pallidum were extracted. Five independent machine learning classifiers [Support Vector Machine (SVM), Stochastic gradient descent (SGD), random forest (RF), quadratic discriminant analysis (QDA) and decision tree (DT)] were trained on the training set, and validated on the testing. Delong test was used to compare the performance of different models.

**Results:**

Our VB-net could automatically identify and segment the brain into 109 regions. 2,264 radiomics features were automatically extracted from the billateral thalamus, caudatum, putamen or pallidum of each patient. After four step of features dimensionality reduction, Delong tests showed that the SVM model based on combined features had the best performance, with AUCs of 0.988 (95% CI: 0.979 ~ 0.998, specificity = 91.1%, sensitivity =100%, accuracy = 89.4% and precision = 88.2%) and 0.976 (95% CI: 0.942 ~ 1.000, specificity = 100%, sensitivity = 87.1%, accuracy = 93.5% and precision = 88.6%) in the training set and testing set, respectively. Decision curve analysis showed that the clinical benefit of the line graph model was high.

**Conclusion:**

The SVM model based on combined features could be used to diagnose PD with high accuracy. Our fully automatic model could rapidly process the MRI data and distinguish PD and HCs in one minute. It greatly improved the diagnostic efficiency and has a great potential value in clinical practice to help the early diagnosis of PD.

## Introduction

Parkinson’s disease (PD) is a complex and progressive neurodegenerative disorder characterized by an insidious onset, high incidence, and significant disability rate (1, 2). It poses a serious threat to the physical and mental health, as well as the overall quality of life, of middle-aged and elderly individuals (3). Its primary clinical manifestations include motor symptoms such as tremors, muscle rigidity, bradykinesia, postural instability; non-motor symptoms such as sleep disorders and olfactory dysfunction; autonomic nervous system dysfunction; cognitive impairment; and psychiatric disturbances (4). Currently, more than 6 million people worldwide suffer from PD, and the number is expected to further increase, bringing a huge burden to families and the society (5).

The rapid and accurate diagnosis of PD is of great significance for targeted treatment, prevention of disease progression, improvement of quality of life and overall prognosis (6). At present, the diagnosis of PD still relies on subjective clinical symptoms (7). Objective diagnosis methods are urgently needed in clinical practice. As a medical imaging technique, MRI has the advantages of non-invasive, non-radiation exposure, and high-resolution capabilities, making it widely used in the diagnosis and staging of neurological diseases (8, 9). Previous studies have identified alterations in both the structure and function of the brain in individuals diagnosed with PD (10, 11). Vogt et al. (12) discovered that the cingulate cortex played a crucial role in identifying new biomarkers for patients with early PD. Nyberg et al. (13) found a significant increasing in the volume of bilateral hippocampal and right nucleus accumbens of PD patients. Kassubek et al. discovered a significant increasing in the gray matter volume of the ventral medial thalamic nucleus on the contralateral side of the tremor limb in patients with PD. Furthermore, they observed a positive correlation between changes in thalamic gray matter volume and tremor amplitude (14). However, these studies mainly focused on macroscopic changes of the brain of PD patients, but overlook the small structural changes.

Radiomics, proposed by Lambin et al. (15), is a new field of computer-aided imaging diagnosis that assists in diagnosing and differentiating diseases by quantifying subtle information in medical images that is difficult to evaluate with the naked eye. Tupe-Waghmare et al. (16) had extracted radiomic features from the subcortical structure, cerebellum, brainstem, and used a random forest machine learning model to identify PD and HCs with an accuracy of 70%. Tafuri et al. (17) used Freesurfer software to extract radiomics features from the subcortical nucleus and used SVM model to distinguish PD and HCs patients, achieving an AUC (area under the receiver operating characteristic curve) of 0.77. However, these methods mostly showed low accuracy and were time-consuming, taking approximately 4 h to process a patient. These shortcomings limited its clinical application. In our previous study, we had developed a CNN-based artificial intelligence model for the automatic segmentation and measurement of the whole brain (109 brain regions), and the entire segmentation and reconstruction process took less than half a minute (18). In this study, we used this network to quickly segment and analyze the radiomics characteristics of the cerebral cortex and neuclei, and try to establish artificial intelligence models to help distinguish PD and HCs.

## Materials and methods

### Participants

All PD patients included in this study were sourced from the PD Progression Initiative (PPMI) database. The PD subjects in the PPMI were newly diagnosed patients who were not receiving any medication. The clinical diagnostic criteria for PD were based on the Movement Disorder Society guidelines. For the most up-to-date information, please visit https://www.ppmi-info.org/. The T1-weighted MR images were obtained using a 3 T Tesla scanner manufactured by Siemens. The imaging parameters were as follows: Acquisition Type = 3D; Flip Angle = 9.0 degree; field of view (FOV) = 256 × 256; matrix = 256 × 256; TR = 2300.0 ms; TE = 3.0 ms; Slice Thickness = 1.0 mm; interslice gap = 0 mm. A total of 155 PD patients and 154 HCs were enrolled in the study. The HC subjects and PD patients were matched for age and sex. All patients were randomly assigned to a training set and a testing set in a ratio of 8:2.

### Brain subregions segmentation

The brain subregions segmentation module was implemented using a deep learning algorithm based on a 3D VB-NET network. The data preprocessing module performed a series of operations, including rotation, resampling, resizing, skull stripping, image non-uniform correction, histogram matching, and gray-scale normalization, on the MRI images used for training and testing. All images are standardized to the size of 256*256*256*1 mm^3^ in the standard Cartesian LPI coordinate system, and the gray-scale range was within the interval (−1, 1). The network training module used an end-to-end deep convolutional neural network, taking each sample image and its corresponding brain substructure partition atlas as the training sample. The sample image was the network input, and the output label was the brain atlas correspondent to the sample image. The network parameters were adjusted according to the difference between the output brain division and the actual brain division, and the training continues until the network basically converges and the output label image was substantially consistent with the partition image corresponding to the sample. In the overall network training process, a coarse-to-fine cascading segmentation strategy was used, simplifying the complexity and difficulty of the brain segmentation problem by step decomposition, providing extra information to the lower level network by the upper level network to enhance the network’s segmentation performance, and achieving fine segmentation of the large brain region, medium brain region, and brain substructures on a stage-by-stage basis. The model was constructed based on 1,800 subjects and evaluation showed an averaged 0.92 Dice overlap with ground truth on 295 subjects. Detailed segmentation process information was descripted in our previously published literature (18).

**Figure 1:**
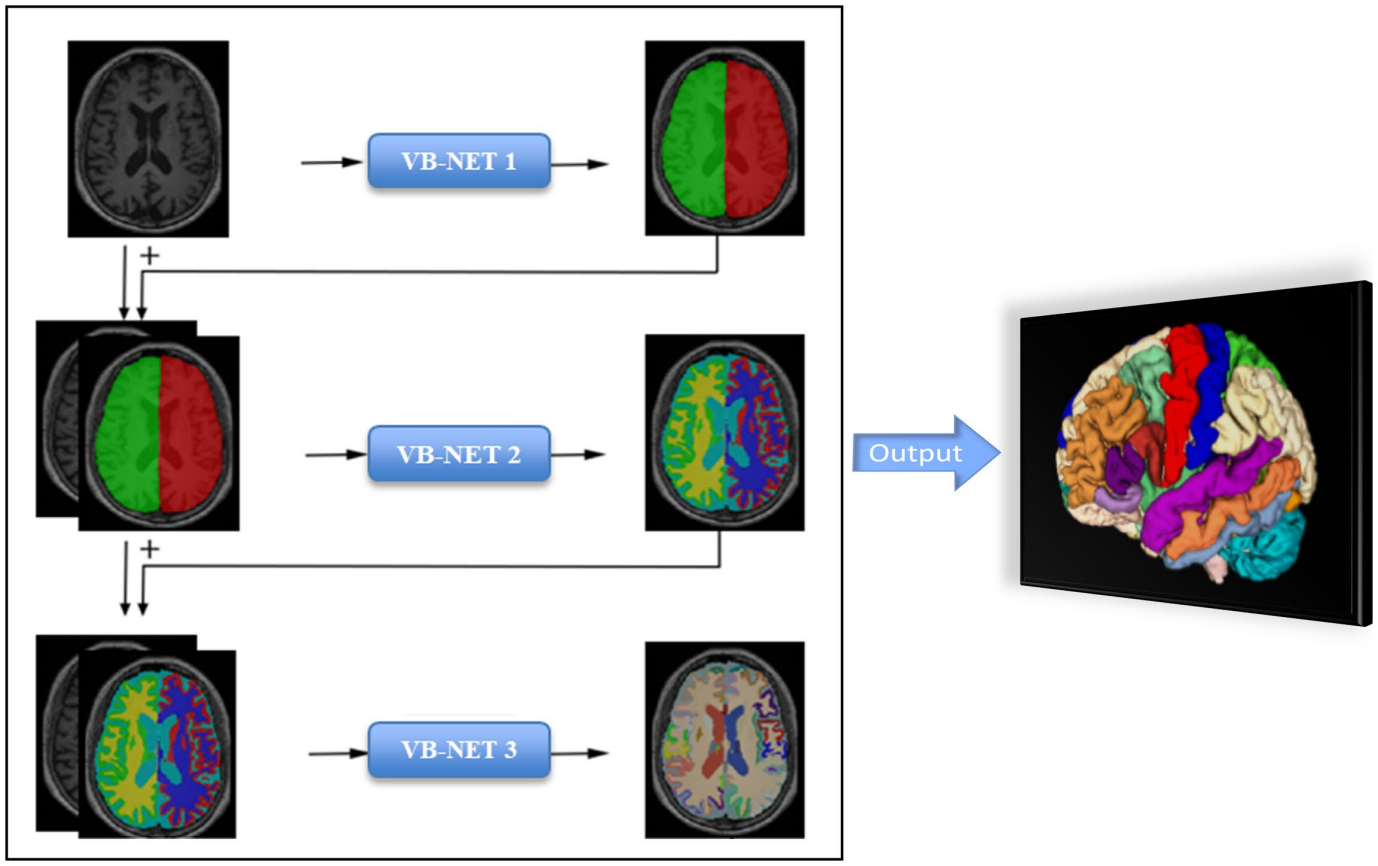
The flow chart of Cascading neural network framework.

The entire brain was automatically divided into 109 subregions, which included 22 subregions in the temporal lobe, 20 subregions in the frontal lobe, 12 subregions in the parietal lobe, 8 subregions in the occipital lobe, 8 subregions in the cingulate gyrus, 2 subregions in the insula, 12 subcortical neuclei, white matter structures, ventricles, cerebellum structures and other structures (Supplementary material 1). The segmentation process took less than half a minute for each patient. The flow chart of brain subregions segmentation was shown in Figure 1.

### Features extraction and dimensionality reduction

The volume of 109 brain subregions of each patient was automatically extracted through the deep learning model. 2,264 radiomics features were automatically extracted from the billateral thalamus, caudatum, putamen or pallidum of each patient. There were 18 first-order statistics and 14 shape features, which reflect the shape and size of the region accurately. Texture features included 21 Gray Level Co-occurrence Matrix (GLCM) features, 16 Gray Level Run Length Matrix (GLRLM) features, 16 Gray Level Size Zone Matrix (GLSZM) features, 5 Neighbouring Gray Tone Difference Matrix (NGTDM) features, and 14 Gray Level Dependence Matrix (GLDM) features. The high level features were obtained through 24 filters (including Box Mean, additive Gaussian Noise, binomial blur, curvature flow, Box-Sigma, normalization, Laplace Sharpening, discrete Gaussian, mean, speck noise, recursive Gaussian, Shot Noise and LoG with sigma values of 0.5, 1, 1.5, and 2), as well as wavelet transformations (LLL, LLH, LHL, LHH, HLL, HLH, HHL, and HHH). All radiomic features were then normalized using z-score normalization, and the reproducibility of these features was assessed using a pipeline that adheres to the recommendations of the Image Biomarker Standardization Initiative.

The relief and least absolute shrinkage and selection operator (LASSO) method were used to select the most robust features. Hyperparameter for LASSO was evaluated using stratified 5-fold cross-validation-based grid search method on the training set. The parameters that provided the highest cross-validation AUC was selected.

### Models building and evaluation

**Figure 2:**
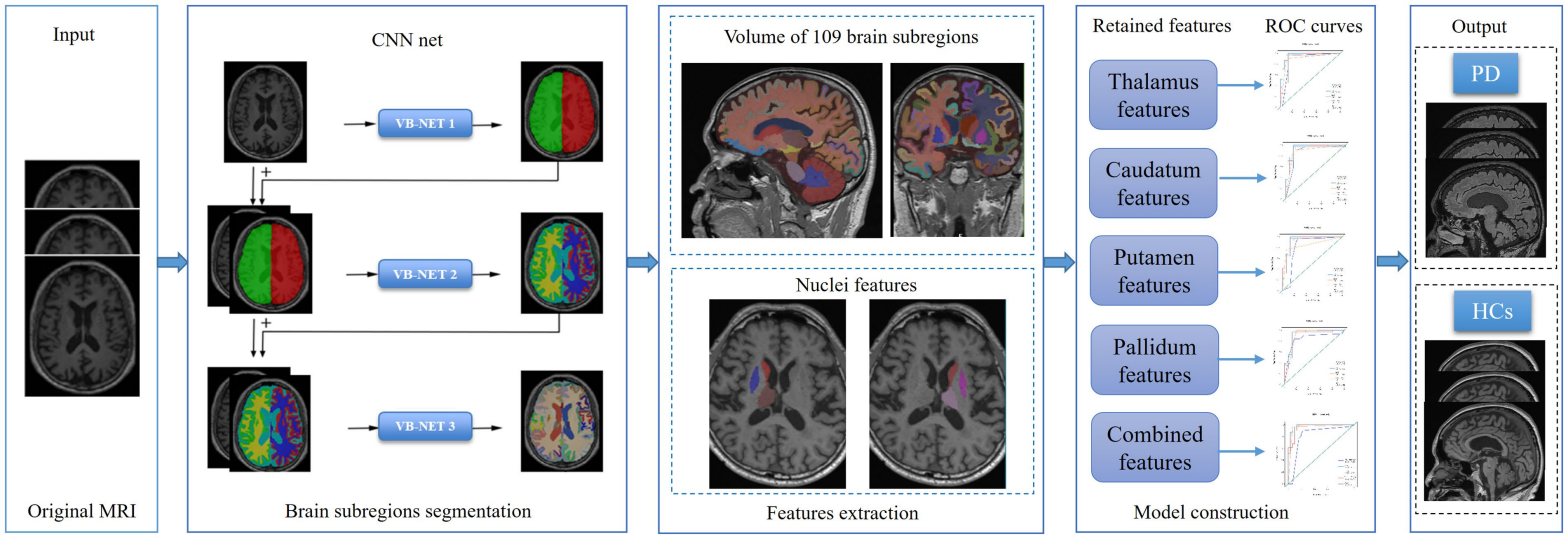
The flow chart of brain subregions segmentation, features extraction, features dimension reduction and models construction.

Based on the retained features, five independent machine learning classifiers, including Support Vector Machine (SVM), Stochastic gradient descent (SGD), random forest (RF), quadratic discriminant analysis (QDA) and decision tree (DT) algorithm were trained on the training set, and validated on the testing set. The performance of classifier models on the test subset was evaluated by the mean and 95% confidence intervals (CI) of the accuracy, sensitivity/recall, specificity, and precision based on a case probability cut-off value of 0.5, as well as the F-score metric and area under the receiver operating characteristic curve (AUC). The calibration curve was used to evaluate the calibration of the model, and DCA was used to evaluate the clinical applicability of the predictive model. The flow chart of our study was shown in Figure 2.

### Statistical analysis

Statistical analyses were performed with R (version 4.0.4) and Python software. Mann Whitney U test or Student’s t test was used for the continuous variables according to the test of normal distribution. The chi-square test was used to compare categorical variables. Statistical significance was indicated by a two-tailed *p* value <0.05. Delong test was used for the comparison of different models.

## Results

A total of 155 PD patients were enrolled in the study, including 99 males and 56 females. Meanwhile, 154 HCs were included, including 99 males and 55 females. There was no significant difference in age and gender between the two groups. The demographic and clinical features of PD and HC were shown in Table 1.

**Table 1:** Clinical and neuropsychological characteristics.

Variable	PD (*n* = 155)	HC (*n* = 154)	*p*
Age (years)	61.2 ± 9.4	70.5 ± 6.5	0.143
Gender (M/F)	99/56	99/55	0.110
Education (years)	15.3 ± 2.9	16.8 ± 2.2	0.109
UPDRS III_score	20.9 ± 9.1		/
MoCA_score	27.6 ± 2.0		/
Modified Hoehn and Yahr Scale	1.6 ± 0.5		/

Two-sample *t* test was used for continuous variable *p*-value and Chi-square test was used for discrete variable *p*-value. *p values less than 0.05 were considered statistically significant.

**Figure 3:**
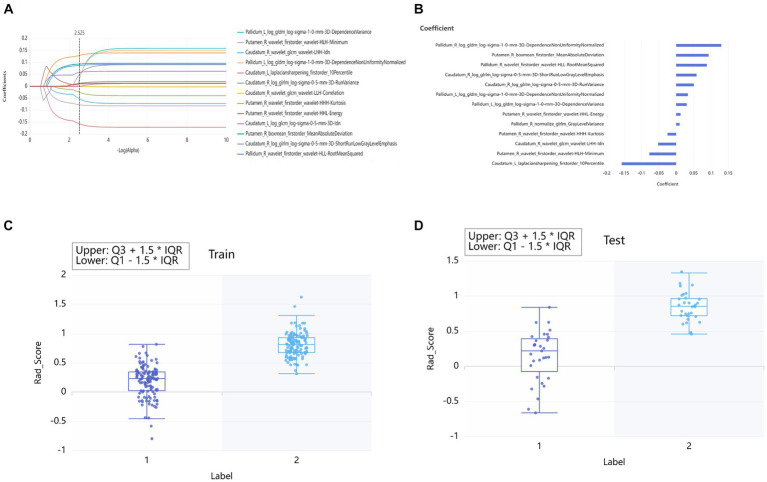
Lasso algorithm for features selection of PD patients and HCs **(A)**. The Lasso path displayed coefficient profiles for radiomic features across the entire range of possible values **(B)**. Rad_Score distribution in the training sets **(C)** and testing set **(D)**. “0” group represented HCs. “1” group represented PD.

In the training set, the volume of 109 brain subregions of each patient was automatically obtained. 100 radiomics features were selected from 4,528 radiomics features of the bilateral thalamus using the relief method, and then 16 optimal features were obtained using the LASSO method. According to the same method, 12, 15, and 10 optimal features were obtained from the caudatum, putamen, and pallidum, respectively. Finally, feature selection of relief method and LASSO were performed again for all the above brain regions together, to obtain the 13 optimal combined features (Figure 3).

**Figure 4:**
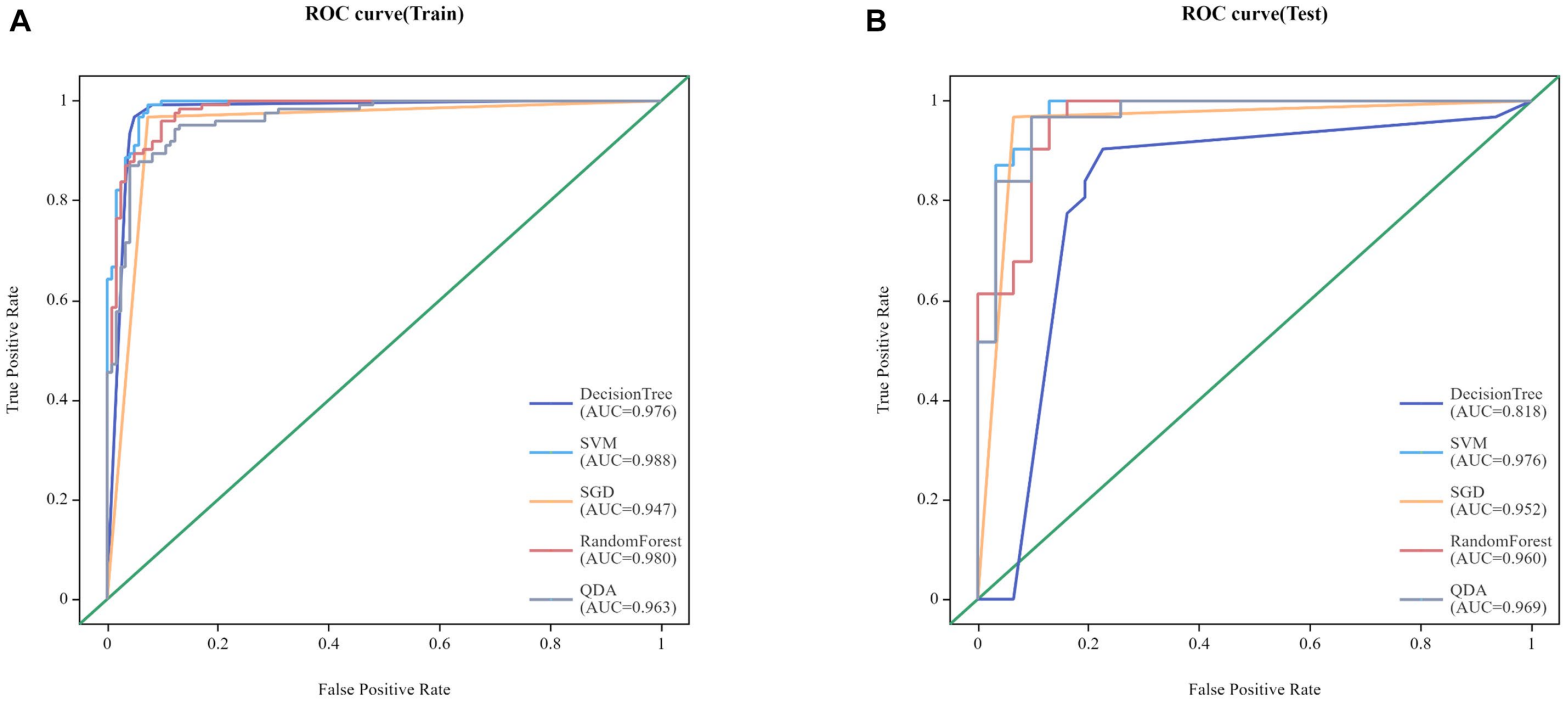
Receiver Operating Characteristic (ROC) curves for SVM, SGD, RF, QDA and DT models in training set **(A)** and testing set **(B)**.

**Figure 5:**
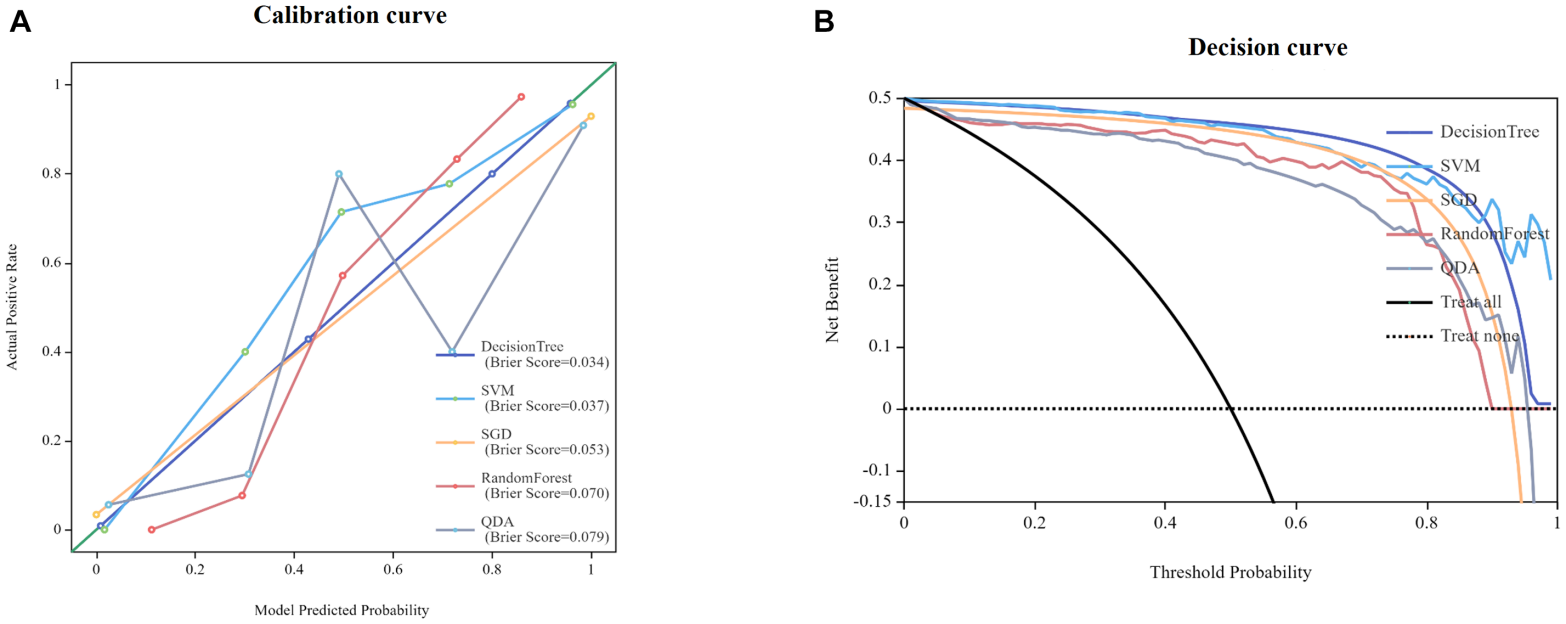
Calibration curves for nomogram goodness of fit **(A)**. Decision curves of different models **(B)**.

Twenty-five models were established based on algorithms of SVM, SGD, RF, QDA, DT and thalamus features, caudatum features, putamen features, pallidum feature and combined features. ROC curves of SVM, SGD, RF, QDA and DT models based on thalamus, caudatum, putamen, pallidum and combined features were shown in Table 2. It was found that the SVM model based on the combined features (5 features from pallidum, 4 features from putamen, 4 features from caudatum) showed the best performance, with AUCs of 0.988 (95% CI: 0.979 ~ 0.998, specificity = 91.1%, sensitivity =100%, accuracy = 89.4% and precision = 88.2%), 0.976 (95% CI: 0.942 ~ 1.000, specificity = 100%, sensitivity = 87.1%, accuracy = 93.5% and precision = 88.6%) in the training set and testing set, respectively (Figure 4). The calibration curve showed a good agreement between the actual and predicted probabilities of the samples (Figure 5A). Decision curve analysis showed that the clinical benefit of the line graph model was high (Figure 5B).

**Table 2:** Results of DT, SVM, SGD, RF, and QDA models on training set and testing set.

	Models	AUC		Sensitivity		Specificity		Accuracy	
		Training set	Testing set	Training set	Testing set	Training set	Testing set	Training set	Testing set
Thalamus features	DT	0.975 (0.957–0.993)	0.892 (0.799–0.985)	0.992	0.968	0.902	0.871	0.947	0.919
	SVM	0.967 (0.945–0.989)	0.970 (0.932–1.000)	0.967	0.968	0.894	0.903	0.931	0.935
	SGD	0.890 (0.851–0.929)	0.903 (0.828–0.978)	0.846	0.903	0.927	0.903	0.886	0.903
	RF	0.958 (0.934–0.981)	0.934 (0.870–0.998)	0.927	0.968	0.862	0.871	0.894	0.919
	QDA	0.866 (0.817–0.916)	0.881 (0.787–0.976)	0.837	0.935	0.813	0.871	0.825	0.903
Caudatum features	DT	0.976 (0.961–0.992)	0.873 (0.772–0.973)	0.911	0.903	0.959	0.839	0.935	0.871
	SVM	0.947 (0.917–0.977)	0.956 (0.907–1.000)	0.919	0.935	0.886	0.871	0.902	0.903
	SGD	0.886 (0.846–0.926)	0.887 (0.807–0.967)	0.87	0.903	0.894	0.871	0.882	0.887
	RF	0.957 (0.935–0.978)	0.937 (0.874–0.999)	0.886	0.903	0.878	0.871	0.882	0.887
	QDA	0.876 (0.828–0.923)	0.921 (0.848–0.994)	0.878	0.968	0.813	0.871	0.846	0.919
Putamen features	DT	0.977 (0.959–0.995)	0.822 (0.699–0.994)	0.951	0.903	0.943	0.774	0.947	0.839
	SVM	0.947 (0.917–0.977)	0.945 (0.885–1.000)	0.935	0.968	0.878	0.871	0.907	0.919
	SGD	0.918 (0.883–0.952)	0.851 (0.762–0.940)	0.935	0.774	0.894	0.935	0.915	0.855
	RF	0.957 (0.935–0.980)	0.939 (0.879–0.998)	0.886	0.935	0.878	0.871	0.882	0.903
	QDA	0.867 (0.817–0.916)	0.912 (0.833–0.990)	0.878	0.935	0.821	0.871	0.85	0.903
Pallidum features	DT	0.958 (0.934–0.981)	0.841 (0.733–0.950)	0.951	0.839	0.886	0.839	0.919	0.839
	SVM	0.941 (0.909–0.972)	0.924 (0.854–0.994)	0.886	0.839	0.87	0.871	0.878	0.855
	SGD	0.849 (0.806–0.893)	0.900 (0.827–0.973)	0.943	1	0.756	0.806	0.85	0.903
	RF	0.947 (0.92–0.973)	0.914 (0.836–0.991)	0.902	0.903	0.854	0.871	0.878	0.887
	QDA	0.877 (0.831–0.923)	0.891 (0.801–0.980)	0.846	0.903	0.813	0.871	0.829	0.887
Combined features	DT	0.976 (0.957–0.996)	0.818 (0.702–0.935)	0.967	0.839	0.951	0.806	0.959	0.823
	SVM	0.988 (0.979–0.998)	0.976 (0.940–1.000)	0.967	0.935	0.943	0.903	0.955	0.919
	SGD	0.947 (0.919–0.975)	0.952 (0.897–1.000)	0.967	0.968	0.927	0.935	0.947	0.952
	RF	0.980 (0.966–0.994)	0.960 (0.917–1.000)	0.959	0.935	0.894	0.871	0.927	0.903
	QDA	0.963 (0.943–0.984)	0.969 (0.931–1.000)	0.927	0.968	0.878	0.774	0.902	0.871

## Discussion

It is well known that PD is a complex progressive neurodegenerative disease with high incidence rate (19). Developing an objective, accurate, and effective method to distinguish PD and HC has become an urgent issue in clinical practice. The changes in the cerebral cortex, deep nucleus, cerebellum and ventricles were found closely related to the occurrence of PD (20). Traditional imaging diagnostic methods were time-consuming, subjective and unable to detect these subtle changes in brain structure (21). Deep learning and radiomics were new fields of computer-aided imaging diagnosis (22), which overcomed the limitations of visual diagnosis and could quantify the subtle information in medical images, providing new hope for the rapid and accurate clinical diagnosis of PD.

In this study, a VB-net network evolved from U-Net network was developed to automatically identify and segment the cerebral cortex, cerebellum, ventricle, and 14 subcortical nuclei in 3D-T1-weighted imaging sequences. 2,264 radiomics features were automatically extracted from each region of the bilateral thalamus, caudatum, putamen, or pallidum. Five machine learning algorithms were applied to classify PD and HCs automatically and an independent verification group was set up to verify the performance of the model. We found among SVM, SGD, RF, QDA and DT classifiers, SVM classifier had the highest classification performance, with an AUC of 0.988 in the training set and 0.974 in the testing set. Our results demonstrated that the application of artificial intelligence technology to analyze raw T1-weighted MRI images could accurately differentiation PD patients from HCs. As a completely objective method, it did not rely on patients’ personal history or doctors’ clinical experience, making it more subjective. To the best of our knowledge, this study achieved the first fully automatic differentation of PD and HCs with high accuracy. Previously, Liu et al. (23) had used a radiomic model based on T2-weighted images of caudate nucleus and putamen to distinguish between PD and HCs, and the AUCs in the training set and testing set were 0.8767 (95% CI: 0.8066 ~ 0.9469) and 0.7143 (95% CI:0.5540 ~ 0.8746), respectively. Their results showed low accuracy and the manual brain segmentation took a long time, greatly reducing its work efficiency and clinical value. Adeli et al. (24) had utilized MRI and Single-Photon Emission Computed Tomography (SPECT) data from 538 subjects in the PPMI database to establish a classification framework, achieving a diagnostic accuracy of 97.5% for distinguishing between PD and HCs. However, the accuracy of their study mainly depended on the SPECT data, while the MRI data only made a small contribution to diagnostic ability. Additionally, the SPECT examination was expensive and involved radiopharmaceuticals that were not easily acceptable by patients (25). In our study, conventional MRI T1 weighted images were used, and all processes were fully automated, resulting in significant clinical significance. Our study was the first to use a deep learning model to automatically detect and segment brain subregions and extract valuable information. Our model could identify PD patients within 1 min and provided high accuracy. With this artificial intelligence system, doctors could quickly and accurately diagnose PD and perform early treatment timely, which could significantly improve prognosis. On the other hand, it could improve medical efficiency, shorten patient waiting time and reduce medical labor costs.

In the optimal model of this study, 13 radiomics features were retained, including 5 features from pallidum, 4 features from putamen and 4 features from caudatum. The 5 features from the pallidum included one Gray-level dependence matrix (GLDM) feature, one Gray-level run length matrix (GLRLM) feature, one first-order feature and two GLDM features. The pallidum receives afferent fibers from the subthalamic nucleus and is the primary region of the basal ganglia that emits efferent fibers (26). It could continuously release inhibitory neurotransmitter γ-aminobutyric acid (GABA), which may cause tremors (27). Hutchison et al. (28) had discovered the presence of neurons that exhibit tremor frequency activity ranging from 4 to 6 Hz in the pallidum, thus supporting the significant role of the pallidum in generating resting tremors in patients with PD. The first-order feature of it reflected changes in the shape and volume of the pallidum (29). Previous studies had demonstrated significant alterations in pallidum volume between PD patients and HCs (30, 31). The loss and degeneration of pallidus neurons, as well as the proliferation of glial cells, could lead to texture changes in MRI images, which were reflected in GLDM and GLRLM. The four features of the putamen were all first-order characteristics, including Mean Absolute Deviation, Kurtosis, Energy, and Minimum. Previous studies had indicated that the putamen appeared to be the first region affected in PD (32). The four features of the caudatum included one first-order feature, one GLCM feature and two GLRLM features. The caudatum is a key substructure of the unique basal ganglia circuit associated with emotional and psychomotor fatigue. It plays a crucial role in the pathological and physiological regulation of PD (33). Previous studies had proved that dopaminergic dysfunction could lead to damage in the caudatum of PD patients, resulting in morphological and pathological changes (34, 35). The first-order features reflected the asymmetry and flatness of the morphology of the caudate nucleus, while the features of GLCM and GLRLM reflected the roughness and heterogeneity caused by pathological changes in the internal structure of the caudate nucleus.

Our study had several limitations. Firstly, this was a retrospective cross-sectional study that did not reflect the dynamic changes of the brain in PD. A prospective longitudinal follow-up study was needed in the future. Secondly, the sample size of PD patients was relatively small and all data were obtained through 3 T Siemens scanners; therefore, the generalization of the model needed to be further verified. In the future, images from multiple research centers were required for independent external validation, so that it could be adapted to a wider range of clinical scenarios. Finally, although we had confirmed that radiomics features have high accuracy in distinguishing PD from HCs, the pathological basis behind them still needed to be further revealed.

## Conclusion

In this study, we established an AI model which could distinguish PD and HCs accurately. It was fully automated and could quickly process the routine MRI data within one minute to obtain accurate results. Our research results have greatly improved the diagnostic efficiency and had a great potential value in clinical practice to help the early diagnosis of PD.

## Data availability statement

The datasets presented in this study can be found in online repositories. The names of the repository/repositories and accession number(s) can be found at: https://www.ppmi-info.org/.

## Ethics statement

The studies involving humans were approved by The PPMI study was approved by an ethics standards committee on human experimentation at each institution. The studies were conducted in accordance with the local legislation and institutional requirements. The participants provided their written informed consent to participate in this study. Written informed consent was obtained from the individual(s) for the publication of any potentially identifiable images or data included in this article.

## Author contributions

ChaL: Conceptualization, Investigation, Methodology, Software, Writing – original draft, Writing – review & editing, Resources. DH: Data curation, Methodology, Software, Validation, Writing – original draft, Writing – review & editing. FW: Methodology, Writing – original draft, Resources. YX: Formal Analysis, Methodology, Software, Supervision, Validation, Writing-original draft. FS: Formal Analysis, Methodology, Software, Supervision, Validation, Writing-original draft. MY: Writing – original draft, Methodology, Software, Validation. JZ: Data curation, Writing – review & editing. CP: Model validation and analysis of Rad_ Score values. JF: Conceptualization, Funding acquisition, Writing – original draft, Writing – review & editing, Data curation, Investigation, Methodology, Resources, Software, Validation. ChuL: Conceptualization, Funding acquisition, Writing – original draft, Writing – review & editing, Investigation, Methodology, Resources.
