# Supplementary material for: Automatic diagnosis of Parkinson’s disease using artificial intelligence base on routine T1-weighted MRI
Source: Front Med (Lausanne). 2024 Jan 5;10:1303501. doi: 10.3389/fmed.2023.1303501 (PMC10797132; doi:10.3389/fmed.2023.1303501)
Supplement: Supplementary file 1 [file Data_Sheet_1.PDF]

Supplementary Material 1:

Region	DK109
1	Precentral_L
2	Precentral_R
3	Postcentral_L
4	Postcentral_R
5	Paracentral_L
6	Paracentral_R
7	Frontal_Sup_L
8	Frontal_Sup_R
9	Frontal_Mid_Rostral_L
10	Frontal_Mid_Rostral_R
11	Frontal_Mid_Caudal_L
12	Frontal_Mid_Caudal_R
13	Frontalpole_L
14	Frontalpole_R
15	Orbitofrontal_Lat_L
16	Orbitofrontal_Lat_R
17	Orbitofrontal_Med_L
18	Orbitofrontal_Med_R
19	Parsopercularis_L
20	Parsopercularis_R
21	Parsorbitalis_L
22	Parsorbitalis_R
23	Parstriangularis_L
24	Parstriangularis_R
25	Insula_L
26	Insula_R
27	Cingulum_Ant_L
28	Cingulum_Ant_R
29	Cingulum_Mid_L
30	Cingulum_Mid_R
31	Cingulum_Post_L
32	Cingulum_Post_R
33	Isthmuscingulate_L
34	Isthmuscingulate_R
35	Hippocampus_L
36	Hippocampus_R
37	Parahippocampal_L
38	Parahippocampal_R
39	Amygdala_L
40	Amygdala_R
41	Caudate_L
42	Caudate_R

43	Putamen_L
44	Putamen_R
45	Pallidum_L
46	Pallidum_R
47	Thalamus_L
48	Thalamus_R
49	Accumbens_Area_L
50	Accumbens_Area_R
51	VentralDC_L
52	VentralDC_R
53	Choroid_Plexus_L
54	Choroid_Plexus_R
55	Ventricle_Lat_L
56	Ventricle_Lat_R
57	Ventricle_Inf_Lat_L
58	Ventricle_Inf_Lat_R
59	Parietal_Sup_L
60	Parietal_Sup_R
61	Parietal_Inf_L
62	Parietal_Inf_R
63	Cuneus_L
64	Cuneus_R
65	Entorhinal_L
66	Entorhinal_R
67	Fusiform_L
68	Fusiform_R
69	Lingual_L
70	Lingual_R
71	Pericalcarine_L
72	Pericalcarine_R
73	Precuneus_L
74	Precuneus_R
75	Supramarginal_L
76	Supramarginal_R
77	Temporal_Sup_L
78	Temporal_Sup_R
79	Temporal_Mid_L
80	Temporal_Mid_R
81	Temporal_Inf_L
82	Temporal_Inf_R
83	Temporalpole_L
84	Temporalpole_R
85	Temporal_Sup_Banks_L
86	Temporal_Sup_Banks_R

87	Transversetemporal_L
88	Transversetemporal_R
89	Occipital_Lat_L
90	Occipital_Lat_R
91	Cerebral_WM_L
92	Cerebral_WM_R
93	Cerebellum_Cortex_L
94	Cerebellum_Cortex_R
95	Cerebellum_WM_L
96	Cerebellum_WM_R
97	Ventricle_3rd
98	Ventricle_4th
99	Pons
100	CSF
101	Optic_Chiasm
102	CC_Anterior
103	CC_Mid_Anterior
104	CC_Central
105	CC_Mid_Posterior
106	CC_Posterior
107	Midbrain
108	Medulla
109	SCP
